# The role of the family doctor in the management of adults who are obese: a scoping review protocol

**DOI:** 10.1186/s40064-015-1647-6

**Published:** 2015-12-30

**Authors:** Elizabeth Ann Sturgiss, Nicholas Elmitt, Chris van Weel, Emily Haesler, Ginny Sargent, Alex Stevenson, Mark Harris, Kirsty Douglas

**Affiliations:** Academic Unit of General Practice, Australian National University Medical School, Canberra, Australia; Radboud University Nijmegen, Nijmegen, The Netherlands; Primary Health Care Research, Australian Primary Health Care Research Institute, Research School of Population Health, ANU College of Medicine, Canberra, Australia; National Centre for Epidemiology and Population Health (NCEPH), Research School of Population Health, ANU College of Medicine, Biology and Environment, The Australian National University, Canberra, Australia; Centre for Primary Health Care and Equity, Centre for Obesity Management and Prevention Research Excellence in Primary Health Care (COMPaRE-PHC), UNSW, Sydney, Australia

**Keywords:** Obesity, Overweight, Adults, Primary care, General practitioner, Family doctor, Primary care physician

## Abstract

**Background:**

The role of family doctors in the management of obesity in primary care will become increasingly important as more of the adult population become overweight or obese. Having a solid understanding of the family doctor’s role as a sole practitioner is important for supporting practitioners in providing patient care and for informing future research.

**Objective:**

The purpose of this paper is to describe a protocol for a scoping review that aims to examine and map the current research base for the role of the family doctor in managing adults who are overweight or obese.

**Methods:**

This scoping review is based on the methodology as described by the Joanna Briggs Institute which involves final consultation with stakeholders. Two reviewers (ES, NE) will be responsible for the iterative development of a search strategy based on the basic initial search terms obesity, doctor and primary care. Black and grey literature will be searched to elucidate any manuscripts involving the family doctor in the management of adults who are overweight or obese. A customised data extraction tool will be used to collect relevant items from each manuscript.

**Results:**

Data extraction will expose the role family doctors are playing in obesity management in all stages of research including recruitment, intervention or as a control group. By looking at a broad scope of manuscripts we will discover the family doctor’s role as portrayed in research, in international guidelines and by peak bodies. We will also determine if there are any gaps in the research base.

**Conclusion:**

This protocol describes a scoping review that will illustrate the supporting international research for the role family doctors are playing in the management of adults who are overweight or obese. Scoping of the international literature will then be translated for Australian primary care.

## Background

The proportion of overweight and obese patients seen in general practice in Australia has steadily increased since 1998. The prevalence of overweight and obese patients increased from 51.8 % (95 % CI 51.2–52.4) in 1998–00 to 58.8 % (95 % CI 58.2–59.5) in 2006–08. It has been estimated from this data that approximately 3 million patients who presented to their GP from 2006–08 were overweight or obese (Valenti 2009).

GPs are usually the first point of care in the Australian health care system. GPs may identify patients who are overweight and not aware, or may be approached by patients for assistance in losing weight. A survey of patients in five NSW practices found that patients identified GPs as having a role is assisting with weight management and 78 % of patients were keen to be regularly reviewed by their GP for weight management (Tan et al. 2006). There is acknowledgement that GPs could be doing more for their patients who are obese and additional supports are needed for them to do this (Jansen et al. 2015; Bennett et al. 2015).

This scoping review aims to identify the role of the family doctor in obesity management by evaluating the current international evidence. It stems from an attempt to perform a systematic review of randomised controlled trials that found only one international trial in which family doctors were the sole practitioner in the intervention (Martin et al. 2006). This broader review aims to determine if this was because randomised controlled trials are not being used to assess the role of the family doctor as a sole practitioner in obesity, or if family doctors as sole practitioners are not being used in interventions for adults with obesity at all. Once the international literature has been evaluated in this scoping review, we will then translate the evidence found for the Australian primary care context.

Current obesity management guidelines strongly recommend the referral of patients to a multidisciplinary team that may include a dietician, exercise physiologist, psychologist, physiotherapist or others depending on the needs of the patient (National Health and Medical Research Council 2013). In some circumstances this multidisciplinary care is not available (e.g. in rural and remote areas), is out of cost range for the patient (Pearce-Brown et al. 2011), involves a long waiting list or is declined by the patient (Tan et al. 2006).

Every health professional should have a clear understanding of their role in the management of adults who are overweight or obese. Helping family doctors to understand what the evidence is for their role allows more open and accurate discussions with patients around possible management options. If the best option according to current guidelines is not available to the patient for whatever reason, the family doctor and patient can then make an evidence based plan for alternative management.

Primary care is defined as that which is accessible as a first entry point into the healthcare system, provides co-ordinated, whole person and longitudinal care that is person-centred (Reeve et al. 2013; van Weel 2014). Person-centredness is defined as the treatment of a patient taking into account their physical health, mental health and social situation. What the patient values and desires for their health remains central to any defined treatment or management process (Reeve et al. 2013).

Family doctors are known by different terms throughout the world including general practitioners (UK, Australia and New Zealand), primary care physician (USA and Canada), family physician or family doctor (USA). They are medical doctors who are trained to have expert generalist skills in patient management (“expert generalist”). In most countries they require extra training above their basic medical degree. The defining feature of an expert generalist is their ability to provide whole-person care and to do this in the context of person-centredness. This translates to being a doctor that can manage all disease and health concerns no matter what body system is affected and being able to do this taking into account the wishes and values of the person at the centre of the management plan (Reeve et al. 2013).

Current systematic reviews of obesity management in adults in primary care do not determine the impact of the different health professionals involved in the intervention (Reeve et al. 2013; Tsai and Wadden 2009; Flodgren et al. 2010). This is important for three reasons:

1. The magnitude of effect of the role of any particular health professional has not been determined
2. As family doctors we cannot assess what specific role we may play if multidisciplinary management is not possible
3. The generalist expertise of the family doctor is not captured and we lose any insight into the effectiveness of this non-fragmented care.

This broad scoping review allows us to synthesise and map the current evidence base for the involvement of the family doctor in obesity management and therefore identify any gaps.

It is well known that existing interventions do not lead to sustained weight loss in the majority of individuals (Fildes et al. 2015). In fact less than 1 % of obese individuals were found to return to normal weight in a cohort study from the UK (Fildes et al. 2015). For policy makers who are involved in decisions related to obesity management in primary care it is important to fully understand the current evidence base for interventions. By broadening our knowledge on the way interventions are currently working we can try to find the “missing link” that may make future interventions more successful.

Our scoping review questions are:

- What supporting evidence do we have for role family doctors play in obesity management for adults in primary care?
- What is the role of the family doctor in managing obesity as a primary risk as supported by the evidence base?
- What do primary care guidelines say about the role of the family doctor? What do peak bodies say about the role of the family doctor? Are these both in line with what is conveyed by current research?

We have searched for similar scoping reviews looking specifically at the role of the family doctor in managing adults who are overweight or obese and none exist to our knowledge (databases searched JBISRIR, Cochrane Database of Systematic Reviews, CINAHL, PubMed, EPPI). A realist review protocol has been published that will review how doctors identify and refer patients who are obese (Blane et al. 2015) but our review will use a different methodology and focus on the role of the family doctor in the management process.

## Methods

### Inclusion criteria

#### Types of participants

This scoping review will consider any manuscripts that discuss the provision of primary health care for adults (18 years +) who have a BMI of greater than 25 (overweight or obese).

#### Concept

Any manuscripts that involve the family doctor in obesity management will be considered including any interventions or discussions of their role. In an intervention trial all stages of family doctor involvement will be accepted whether that be in the recruitment phase, the intervention itself or as a control. This will be regardless of whether or not other health professionals or lay people are involved.

#### Context

This scoping review will consider manuscripts that involve a primary care setting whether in the recruitment phase or during the intervention phase.

#### Types of sources

All sources of information will be included including studies published in peer-reviewed publication (black literature) and non-peer-reviewed (grey) literature. In the grey literature we will search specifically for international guidelines and announcements from peak bodies.

### Exclusion criteria

- Complete text in languages other than English (translated abstracts will be assessed)
- Exclude studies of children (under the age of 18 years) and family interventions where the primary target of the intervention is the child
- Exclude if participants recruited/treated only in a tertiary facility
- No publication date exclusion; no type of manuscript excluded.

### Search strategy

Our search strategy will involve three steps as described by the Joanna Briggs Institute methodology for scoping reviews (The Joanna Briggs Institute 2015).

1. An initial limited search of two databases (Medline, CINAHL) with “[(obesity) and doctor] and primary care” will be performed.
  - We will analyse the text words in title, abstract and index terms of relevant studies found to compile a list of relevant search terms.
2. Then using all identified keywords and index terms we will search across all of the following databases:
  - Medline, CINAHL, Cochrane, PsycInfo, DARE, Scopus
  - New York Academy of Medicine Grey Literature Report, Open Grey
  - International guidelines for primary care via the World Organisation of National Colleges, Academies and Academics Associations of General Practitioners and via national primary care colleges’ websites (English and non-English speaking—Australia, UK, USA, New Zealand, The Netherlands, Denmark, Finland, Estonia, Slovenia, Belgium, Spain and Portugal).
3. Finally we will search the reference lists of all identified material to identify further material of relevance.
  We will contact authors of primary studies or reviews for further information as appropriate.

Lists of articles will managed with reference software and duplications will be removed. The title, abstracts and keywords of all articles will then be reviewed by two independent reviewers (LS, NE) to determine whether they meet our inclusion criteria. In cases of uncertainty the entire article will be reviewed and in cases of disagreement a third author (KD) will be consulted.

We will then review the full publication for any articles that meet our inclusion criteria.

### Assessment of methodological quality

A formal assessment of methodological quality is not a typical feature of a scoping review. No formal assessment of quality will be included in our scoping review.

### Extraction of the results

Data will be extracted using a customised data extraction form based on the TIDieR framework (Hoffmann et al. 2014) that will trialled between the two reviewers prior to full data extraction. The data extraction will be modified as needed for different manuscript types (e.g. research, opinion, guidelines). The two reviewers will then extract the data independently with any conflict being resolved with discussion with a third reviewer. The data extraction form will contain the following information:

1. Author
2. Year of publication
3. Country of origin
4. Aim of the research as described by the authors
5. Population and sample size, including any co-morbidities
6. Methodology
7. Intervention/comparator (if applicable)
8. Duration of intervention (if applicable)
9. Outcomes and how these were measured
10. Key findings as applicable to this scoping review:
  - In what way was primary care involved (recruitment/intervention/control/other)?
  - How was a doctor involved? (recruitment/intervention/control/other)?
  - What skills were required of the family doctor? (identification/nutrition/physical activity/behavioural intervention/medication/other)
  - Did the intervention meet the definition of primary care:
    i. first point of entry? (the patient could access this service without a specific referral)
    ii. whole person case? (is the health of the person as whole considered?)
    iii. person centred care? (are the values and beliefs of the patient taken into account in the context of their physical, mental and spiritual health?)
    iv. longitudinal? (is this delivered in a fashion that could be continuous, or intersect with continuous care?).

As is customary with scoping reviews, the data extraction template will be reviewed as necessary as the data extraction proceeds. This will be determined during weekly meetings of the two reviewers.

### Presentation of the results

The results of our search strategy will be presented as a PRISMA flow diagram as per convention. The data extraction will be presented as a table with the following headings which may be refined as the scoping review proceeds: Year, Country, Aim(s), Methodology, Intervention, Family Doctor’s Role.

A narrative synthesis of the included studies will allow for direct discussion of the scoping review objectives. We will identify any gaps in the literature and discuss any implications for practice or future research.

### Consultation

Our results will be presented to local Australian stakeholders to assess whether the findings resonate with what they know and experience of primary care weight management programs. This will be done via a public forum where the results will be presented and a discussion panel involving our research team will be conducted. Stakeholders who will be specifically invited will include local GPs and primary care nurses, academic GPs, policy makers and tertiary weight management clinicians. After the forum an opportunity to give direct feedback to the research team via email will be given. This will also facilitate knowledge exchange between clinicians and policy makers in the area of obesity in primary care.
